# Bullying and Emotional Problems in Pupils from 11 to 13 Years Old: Joint Detection through Self-Report

**DOI:** 10.3390/ijerph192114306

**Published:** 2022-11-02

**Authors:** Ana-Isabel González-Contreras, José-Luis Ramos-Sánchez

**Affiliations:** 1 Faculty of Education and Psychology, University of Extremadura, 06006 Badajoz, Spain; 2 Council of Education and Employment, 06810 Mérida, Spain

**Keywords:** bullying, emotional problems, peer violence, school coexistence

## Abstract

The objective of this study was to adapt and make available a valid instrument based on a joint questionnaire (self-report type) to detect the risk of bullying and emotional problems in pupils aged from 11 to 13 years. The questionnaires used were that of Spain’s Ombudsman (Defensor del Pueblo) to detect the risk of becoming a victim of bullying and an adaptation of Spain’s CECAD to assess the risk of emotional problems. The participants were 1077 gender-matched subjects enrolled in the 6th year of Primary Education (*n* = 467) and the 1st year of Lower Secondary Education (*n* = 610) from 19 schools in the Region of Extremadura. High reliability was obtained in both questionnaires, as well as a significant relationship between bullying and emotional problems (0.36). The scales place the pupils at either a certain risk level (mild, moderate, or severe) or no risk. The study concludes with the description of four situations deriving from the cross relationship between victimization and the pupil’s emotional problems: (1) no risk of bullying and no risk of emotional problems (73.2%), (2) risk of bullying but no risk of emotional problems (11.1%), (3) no risk of bullying but risk of emotional problems (9.4%), and (4) risk of bullying and risk of emotional problems (6.3%).

## 1. Introduction

For some decades, the phenomenon of bullying has been a matter of concern for education systems, and proof of this lies in the actions that schools implement as promoted by their Public Administrations. Bullying occurs in all contexts and countries, and takes on different manifestations depending on the victims’ and aggressors’ characteristics and on the sociocultural context.

A one-time insult cannot be considered bullying, but when it occurs repeatedly a process begins that can last a long time, with the consequent psychological suffering of the victim [1]. On numerous occasions, the victim keeps quiet about the bullying, accepting a position of inferiority to the aggressor whom they dare not denounce or confront. Often neither the teachers nor the families are aware of the situation, which makes detection even harder. Nonetheless, witnesses (peers of the victim) are frequently aware of the facts, but dare not inform the adults for fear of also becoming victims or of being excluded from their social group.

Abuse among pupils has a negative effect in childhood and adolescent behaviour, and requires education systems to take action to prevent and deal with it. Any schoolchild can be a victim of bullying when they become the focus of attention of a group or individual aggressor who abuses physically, verbally, and/or psychologically. The idea needs to be rejected that it is “just kids playing”, and the possibility must be considered of its having very negative consequences for the victim’s appropriate emotional development, and that in the most serious cases it can lead to suicide. For this reason, teachers should be offered instruments that are easy to use and interpret without the need for specialized training in risk detection processes, and know that they can count on other professionals if required (psychologists, pædagogues, school counsellors, …).

Olweus [2,3] dealt with dealt with giving a precise definition of what bullying is—that it occurs when an individual or a group is the victim of frequent negative actions by one or more others. The victims live in a situation of inferiority, with a clear physical and/or mental inequality being observed relative to the aggressors whose intent is to hurt or cause them discomfort or physical and/or psychological harm. Similar definitions have been accepted by the scientific community [4,5,6]. Especially relevant seems to be the work of Urra & Reyes [7]. Based on an extensive analysis of the definition of bullying in twenty-eight countries on five continents, it reaches a similar definition in which abuse, the helplessness the victim suffers, and their exclusion and loneliness stand out. However, some studies have shown that when pupils are asked to explain what bullying is, only 1.6% of them express intentionality, 2% refer to the repetition of the aggressive behaviour, and 26% point to inequality of power, contrasting with the 92% who highlight the negative actions taken on the victim [8]. This suggests that the victims’ perceptions of abuse could be influenced by personal, social, and cultural differences.

Although the school environment is the most frequent context in which bullying is detected, some studies show that victimization experiences are better perceived in the family environment [9,10,11]. Younger pupils more often tell about their experiences to their families than to their teachers [12]. Bullying related to close associates (siblings and peers in childhood and adolescence) has also been shown to predict a greater likelihood of mental health problems in early and late adulthood, such as depression, suicidal ideation, and self-harm [13,14,15,16]. Similarly, the study by Lazo [17] in which pupils from 12 to 17 years of age participated showed that the percentages of victimization, both psychological and physical, due to bullying increase in the school environment as the exposure to domestic violence increases. The family is a very important referent in the detection process. For this reason, it is necessary to contrast the information obtained from the questionnaires (self-reporting) with that provided by the family on the characteristics of the pupil’s situation within the family.

The numerous programs that have been launched in different contexts and from different points of view to prevent bullying constitute a sign of the interest in this subject. For example, the Olweus Bullying Prevention Program (OBPP) [18] was designed to reduce bullying and achieve better relationships among pupils in primary, lower secondary, and higher secondary education. In Finland, there is a prevention, intervention, and follow-up program called KiVa which, unlike other programs, is characterized by the fact that the prevention is based on the behaviour of the witnesses. There is significant evidence of the reduction of cases of bullying in those countries where it has been implemented [19].

In Spain, programs such as Tutoring Among Peers (Tutoría Entre Iguales, TEI) [20] is one of the most commonly applied in the school environment since it adapts to the organization of the school and only requires affordable financial outlay and personal resources. Its objective is to facilitate the process of adaptation and social integration of pupils joining the school for the first time. They will have a referent who will be their classmate-tutor, which thus reduces their own insecurity in the unknown organizations and compensates for the imbalance of power that exists between the aggressor and the victim.

With respect to the incidence of bullying, reports from UNESCO [21] and Save the Children [22] highlight an increase that places Spain with 8.1% of occasional bullying and 1.2% of frequent bullying, with differences between regions of the country. In recent years, this incidence has been aggravated by cyberbullying, a form of bullying which uses digital resources and causes the victim psychological damage [23,24]. It has been observed [25] that the frequency of bullying is greatest in the years from 6th of Primary to 1st of ESO (“Educación Secundaria Obligatoria”, Spain’s term for lower, i.e., obligatory, secondary education), and that this is therefore the best time to detect the said problem in schools [26].

With regards to the relationship between bullying and emotional problems, the study by Fuentes [27] showed that victims’ psychosomatic problems were significantly greater in girls, but that in both boys and girls they were associated with sadness, headaches, or stomach ache with no known cause. Peña & Aguaded [28] showed the existence of significant correlations between emotional intelligence and well-being, bullying and emotional intelligence, and well-being and bullying, which justifies the need for detection, prevention, and the implementation of programs to improve emotional well-being and school coexistence from the beginning of adolescence.

A common aspect of bullying prevention and intervention programs is the use of questionnaires to detect these situations. Since Olweus [29], the interest in creating instruments to detect both bullying and violence in general has increased considerably, not only directed at pupils but also at teachers and parents [30]. González & Molero [31] analysed the characteristics of fourteen questionnaires for the detection of bullying and cyberbullying in the Spanish-speaking community. They selected these questionnaires from articles indexed in scientific journals from 2011 to 2021, with quantitative cut-off methods and with samples of adolescents. Seven of them are aimed at detecting partner violence and another seven at detecting school violence. A common aspect is that the data collection is carried out in schools, as it this is the environment in which adolescents develop their social activities most extensively, and is a relatively easy and organized context to apply and collect the samples. Likewise, the studies by Gutiérrez [32] and González & Arrimada [33] describe and compare different instruments used in recent years, highlighting the importance of measuring not only the type of violence involved, but also other emotional and psychosomatic factors that are difficult to observe. Nonetheless, although the questionnaire has been the most frequent and effective instrument in the detection process, the mixed model sociometric approach (qualitative-quantitative, observation, and questionnaire) [34] has also demonstrated its effectiveness.

Avilés [35] not only showed the importance of interviewing the victim as a technique to obtain information since thanks it makes it possible to determine the seriousness of the situation, but also found that it is common for the victims to withhold information, even from their family, out of fear or shame. However, to help the adult plan an upcoming intervention, it is essential to know the victim’s opinion, and assess how they perceive and interpret what is happening. In addition, the opinion provided by the aggressor and information collected from the observers or witnesses of the situation constitute ways that, when used together, will be very effective in obtaining objective information on the scope, intensity, and characteristics of the abuse in the case of it occurring.

The objective of this study was to adapt and make available a valid instrument based on a joint questionnaire (self-report type) to detect the risk of bullying and emotional problems in pupils aged from 11 to 13 years. The results will allow different scores to be obtained to assess the risk of becoming a victim of bullying and/or of suffering emotional problems that may or may not derive from the violence itself. The fact of measuring both variables at the same time is due to their significant relationship, since a pupil who is a victim of bullying is quite likely to have emotional problems deriving from the perceived violence.

## 2. Materials and Methods

### 2.1. Sample

The sampling design was carried out taking into account the main characteristics of the schools and pupils of the Region of Extremadura: ownership (public, private), education level (6th Primary Education and 1st ESO—between 11 and 13 years old, mean 12 years and 6 months), and the gender of the participants (boys and girls). An initial sample of 1283 subjects was obtained. After the data had been cleaned and the incomplete questionnaires eliminated, the valid sample left was 1077 subjects—549 boys (51%) and 528 girls (49%), 467 in 6th of Primary (43.4%) and 610 in 1st of ESO (56.6%), from 15 public (*n* = 899, 83.5%) and 4 private (“concertado”) schools (*n* = 178, 16.5%). Taking into account the possible sociocultural differences with other Regions of the nation, we dare not generalize the results to the rest of Spain.

### 2.2. Design and Procedure

This is a descriptive, cross-sectional, and quantitative investigation. Questionnaires were used in the form of self-reports that were provided to the schools by the research team. The purpose and objective of the study was explained to the Department of Education & Employment of the Junta de Extremadura regional government, who authorized entry to the schools, and acceptance by the management teams of the schools and the families was requested. The questionnaires were printed out and applied by the person responsible for the school’s educational orientation or by the teacher-tutor of the group-class. To guarantee adequate application, the necessary instructions were given and any doubts that arose were resolved. The questionnaire was anonymous. The time for the application and collection of the completed questionnaires was approximately six months from when the school was contacted until all the questionnaires had been collected by the research team.

### 2.3. Instruments

To gather the information, two questionnaires were used. Bullying among peers was evaluated with the Ombudsman-UNICEF Questionnaire [36], and the Clinical Educational Questionnaire: Anxiety & Depression (CECAD) [37] was used to detect emotional problems.

The Ombudsman-UNICEF questionnaire is one of those most used to evaluate bullying in Spain. With this instrument, questions can be asked from different points of view (as victims, aggressors, witnesses, and teachers) and various types of aggression are identified (verbal, indirect physical, direct physical, social exclusion, threats, and sexual bullying with actions or comments). In our study, the questionnaire was aimed exclusively at detecting victims of bullying. The pupils responded to 13 questions assessing the perceived frequency with which the situations occur, expressed on a scale of 1 to 4: 1 (never), 2 (sometimes), 3 (often), 4 (always). From the sum of the scores for each item, a value was obtained that quantifies the seriousness of the bullying situation. In the report issued in 2007, there were no indicators of reliability or validity, although numerous research studies have used this instrument and its effectiveness has been demonstrated in the study of bullying in the school environment. The total score of the scale ranges between 13 and 52 points.

For its part, the CECAD questionnaire allows the emotional situation of subjects from 7 years old to be evaluated (71.7% of the original normative sample were between 7 and 12 years old). It provides essential information about two common emotional disorders, anxiety and depression, as well as other clinical aspects such as worthlessness, irritability, problems of thinking, and psychophysiological symptoms. From the 50 items that make up the original complete test, its authors demonstrated adequate reliability indices (between 0.79 and 0.91) on the different scales, as well as on the complete scale (Cronbach’s *α*, 0.94). Although the complete test was applied in our study, the adapted version has the 16 items which, according to statistical criteria, best detect pupils’ emotional problems at these ages. The response to each item of the questionnaire is on a scale from 1 to 5 representing the frequency of occurrence expressed by the subject: 1 (never), 2 (almost never), 3 (sometimes), 4 (almost always), and 5 (always).

### 2.4. Statistical Analysis

The condition of polytomy of all the items in both questionnaires was considered. Analyses were performed using the statistical package SPSS v.25 and, complementarily, the application Factor Analysis (vn 12.01.02) [38] for some analyses that will be described in the corresponding section, especially for the selection of items in the adaptation of CECAD following the proposals of Ferrando [39]. Reliability studies were carried out (standardized Cronbach’s *α* and ordinal McDonald’s *ω* indices). These were completed with a measure of sampling adequacy index (MSA), arithmetic mean, standard deviation (SD), skewness (Sk), and kurtosis (Ku) for each item.

The Solomon method [40] was used to evaluate the equivalence of the subsamples, and a “community ratio” index (CRI) was obtained. The closer this index is to unity, the greater the equivalence of the samples and the more robust the results using the full sample. This method is of interest because it optimally divides the total sample into two equivalent halves and guarantees the representativeness of the sub-samples, demonstrating control of all possible sources of variance.

Factor analyses of the two questionnaires were done independently in order to identify their factorial structure, using the unweighted least squares (ULS) method with promin rotation (an oblique rotation, useful in a broad set of data and with related factors). The suitability of the sample and the adequacy of the factor analyses were verified using the Kaiser-Meyer-Olkin (KMO) test, the Bartlett sphericity test, and the root mean squared error of approximation (RMSEA). Insignificant factor loadings (coefficients less than 0.30) were eliminated.

The bullying victim questionnaire has 13 items and required no adaptation, while the original CECAD questionnaire (50 items) required a factor analysis of the items that considered a linear model. The intention was to have a brief instrument to be filled in as part of a detection process in the context of a school or a primary healthcare consultancy. For this reason, the items best representing the complete application of the test were selected, taking into account two statistical criteria: eliminate MSA values less than 0.50 and, in the emotional problems questionnaire, select only those items with MSA values very close to or greater than 0.90.

Comparative studies were done for gender, education level, and ownership. Effect size analyses were planned using the G*Power application (vn 3.1.9.7) [41].

In order for the results to be applicable to the school context, the main overall descriptive statistics of each scale and the critical scores identifying the pupil’s emotional problems and/or self-perception as a victim of bullying were determined. In both scales, a criterion of 85th percentile and above was used to detect pupils at risk in the corresponding aspect, and different levels of intensity were considered (mild, moderate, severe). Pupils whose scores were below that percentile were considered to be without risk. The reason for the choice of this threshold was that, in a study by González Contreras [42] which applied the same Ombudsman questionnaire and differentiated between mistreatment (aggression occurs “sometimes”) and bullying (aggression occurs “often” and “always”), the joint mistreatment + bullying percentage was 17.8%, so that it is reasonable to place the critical point of risk at a percentile above the 85th.

In addition, the relationship was studied using two procedures—calculating the Spearman correlation coefficient (*ρ*) between the total scores on the bullying detection and the emotional problems scales, and performing comparative studies.

## 3. Results

### 3.1. The Solomon Method, and the Items’ Descriptive Statistics

The preliminary analysis that the Factor Analysis application offers is the SOLOMON method to divide the total sample into two equivalent samples. We verified that in the two questionnaires the two random subsamples were very similar to each other. The IRC values were close to unity, and the KMO values in each subsample of each questionnaire were also close to unity and exceeded the minimum acceptable value of 0.75, especially in the emotional problems questionnaire which were obtained from the selection of 16 items, with which we shall obtain quite stable results (Table 1).

In the bullying victim questionnaire, we found that the most frequent bullying actions were those related to insults (M = 1.54), name calling (M = 1.53), and bad-mouthing the victim (M = 1.50). The least frequent were threatening with weapons (M = 1.02), sexual harassment (1.03), and using threats to force the victim to do things (M = 1.03). The measures of sampling adequacy (MSA) were all greater than 0.50 and quite close to unity, reflecting the degree to which each item gave the same measure as the test as a whole. The types of aggression were ranked from highest to lowest according to the frequency represented by the mean (Table 2 and Table 3).

### 3.2. Reliability

The reliability of the questionnaires was obtained by two procedures provided by the Factor Analysis application. Taking into account the characteristics of the polytomous items of the scales, the standardized Cronbach’s *α* and McDonald’s ordinal *ω* were the following: bullying questionnaire (*α* = 0.924 and *ω* = 0.925) and emotional problems questionnaire (*α* = 0.931 and *ω* = 0.929). In both cases, the values were very high.

### 3.3. Factorial Structures

In the bullying victim detection questionnaire, the polychoric correlation matrix gave a high KMO value (0.88), showing the suitability of the sample size, as well as the adequacy for factor analysis in obtaining significant values in the Bartlett sphericity test (8974.2, df 78, *p* < 0.0001) [43]. Three factors were obtained that explain 70.3% of the total variance, with the following distribution of variance percentages among them: Factor 1 = 53.1%; Factor 2 = 9.4%, and Factor 3 = 7.8%. The model’s fit was excellent, with an RMSEA of 0.012 (df = 42) being well below 0.05 and a Goodness-of-Fit Index (GFI) = 0.996 [44]. The distribution of the items among the factors is presented in Table 4. The items for each factor are ordered by factor loading. Factor 1 comprises items related to indirect aggression, Factor 2 serious threats and sexual harassment, and Factor 3 behaviour aimed at ridiculing, scaring, excluding, and physically assaulting.

The results of the factor analysis in the detection of emotional problems confirmed the suitability of the sample with a very high KMO coefficient (0.945), and significant values in the Bartlett sphericity test (10,937.9, df 120, *p* < 0.000) [26]. Two factors were obtained that explain 59.8% of the total variance, the case being practically one-dimensional since Factor 1 explains 50.6% of the total variance and Factor 2 explains 9.2%. The model’s fit was excellent, with an RMSEA of 0.037 (df = 89) being below 0.05 (GFI = 0.996). The distribution of the items among the factors as represented by the structure matrix is presented in Table 5. Factor 1 is seen to comprise psychosomatic items, while Factor 2 is made up of negative self-perceptions (sadness, emotional depression, low self-esteem, feeling of worthlessness, and fear).

By having the data from the original (50 items) CECAD completed by the 1077 subjects in the sample, the predictive capacity of the adapted (16 items) questionnaire was verified. Using SPSS’s linear regression analysis, the *R* (Pearson) coefficient between the total scores of the selection of the 16 items (predictor variable) and the total score of the complete questionnaire (dependent variable) was 0.949 (adjusted coefficient of determination = 0.90, standard error of the estimate = 9.304). This result is therefore further evidence for the good fit of the adaptation with the selected items.

### 3.4. Comparative Analyses

Taking into account that the normality condition was not met, the Mann-Whitney U test for two independent groups was applied in all three comparisons made (gender, education level, and ownership).

#### 3.4.1. Gender

The results showed no significant differences by gender, as is reflected in the low values of the effect sizes (less than 0.20) according to Cohen’s interpretation [44,45]. In the bullying detection scale, the values of the arithmetic means are not significant (0.134). They are significant, however, in the emotional problems scale (*p* < 0.05), with the value of the effect size (0.16) and the statistical power (1-*β* = 0.72) being low (Table 6).

#### 3.4.2. Education Level

There were no significant differences by education level in bullying or in emotional problems, as shown by the Mann-Whitney U test. As expected, the effect sizes were very small (Table 7).

#### 3.4.3. Ownership of the School

Table 8 shows that there were no significant differences by ownership in either the victim of bullying or the emotional problems detection scales. With the smallness of the difference in the means and standard deviations between the two groups, the M-W U test *z* values were not significant, and both the effect size and the statistical power were very low.

### 3.5. Descriptive Statistics and Risk Interpretation Tables

The overall values of the total scores of each questionnaire are assumed to represent the position of the subject. For this reason, the sum of the scores is a very stable value that places each pupil at a certain level of risk in their perception both of being a victim of bullying and of having emotional problems. Table 9 presents the most representative descriptive values. Bearing in mind that no significant differences were found in terms of relevant variables (gender, education level, and ownership of the school), the statistics identify the position of a subject in relation to their reference group. The table gives the raw scores corresponding to the 25th, 50th, and 75th percentiles. In the bullying scale, these scores are 13, 15, and 17 respectively, while in the emotional problems scale they are 20, 25, and 33.

The centile score was used to construct the risk tables. The quantitative values and their corresponding interpretations are given in Table 10. The critical score to determine the risk of being a victim of bullying and/or having emotional problems was that of the 85th percentile. For example, on the bullying victim scale, a raw score of 18 and below would be in the “No risk” zone (percentile < 85).

### 3.6. Correlation Studies

Correlation studies between the two scales were made from the total raw scores. Spearman’s bivariate coefficient (*ρ*) was used, obtaining a value of 0.360 (*p* = 0.000) for the correlation between the scores for bullying victim and emotional problems. This corresponds to a moderate level of relationship between the two variables. The effect size obtained with the G*Power application was moderate in accordance with Cohen’s interpretation [28], and the statistical power was perfect (1.00). This fact confirms the trend that pupils who are victims of bullying also present emotional problems.

One observes in Table 11 and Table 12 that, as the score on the emotional problems scale increases so does the score on the bullying detection scale, and vice versa. The lowest scores in emotional problems were for those who obtained the lowest scores on the bullying detection scale (15.51), while the severe risk group were those who obtained the highest mean (20.37).

In comparing the mean in emotional problems with the score obtained on the bullying victim scale, we found that there were no significant differences between the scores in emotional problems when the risk of being a victim was at the mild and moderate levels (centile between 85 and 94), so that these intervals were merged, obtaining the mean of that group.

The frequencies and percentages of the two variables studied (victim of bullying and emotional problems) were crossed based on the risk categorized from simplifying Table 11 and Table 12 (some level of risk –mild, moderate, severe– and no risk). Situation 1 is the most frequent (*n* = 788, 73.2%) and corresponds to pupils who neither perceive bullying nor show emotional problems. Situation 2 is the group that perceives bullying but is not at risk of emotional problems (*n* = 120, 11.1%). Situation 3 corresponds to pupils who show no risk of bullying but do show emotional problems (*n* = 101, 9.4%). Situation 4 is the most serious, and corresponds to pupils who are in the risk zone for bullying and also for emotional problems (*n* = 68, 6.3%).

## 4. Discussion

The phenomenon of bullying is one of the most worrying problems in schools for pupils from the age of 10 onwards, among other reasons because it goes unnoticed. As stated by Varjas, Henrich & Meyers [46], the cause of its invisibility is due to its usually being considered a one-off conflict that precludes teachers and families from looking in depth into the situation. This is why actions of detection and prevention are needed, especially when situations of bullying that begin in the last years of primary education go on to secondary education [47]. To this is added cyberbullying, which increases progressively from 5th of primary education [48], motivated by the widespread availability of mobile phones from 10–11 years in age onwards [49].

From a qualitative approach based on in-depth interviews, Fort concludes that pupils, families, and teachers are aware of the phenomenon of bullying, but its importance has been relativized, and there has been only very superficial implementation of programs [50]. Knowledge of the prevalence and type of bullying and having a detection process available are therefore very important before general programs of prevention and intervention and of aid to the victims can be implemented. The families and teachers, and the aggressors themselves will also need advice and support in this task.

With regard to the types of bullying, our results coincide with the Fundación Mutua Madrileña & Fundación ANAR study [51]: greatest percentage of verbal aggression (between 20% and 37% insults, spreading rumours, and social exclusion) and to a lesser extent physical aggression, theft, and damage to property (between 6% and 20%). That study found that the age of 12 (with an interval between 11 and 14) is that with the greatest frequency of bullying. This is coherent with the age range used in the present study’s detection process. Independently of the type of aggression, the results reported by Garaigordobil & Machimbarrena [52] with a sample of pupils between 9 and 13 years old confirm ours in that those who obtained higher scores in victimization also showed higher levels of stress.

The earliest possible detection, prevention, and intervention should be a constant element in all schools and an objective of coordination with other community services (health, justice, police, …). Victimization can become chronic, and this is prone to develop symptoms of maladaptive behaviour such as becoming victims of family abuse, exclusion, and social anxiety [53]. Likewise, the results of Álvarez Marín et al. [54] show lower self-esteem and more symptoms of emotional problems than in non-victims or bullies.

As no relevant differences were found based on gender, ownership of the school, or education level, this allowed specific and undifferentiated risk tables to be constructed according to these variables. Starting from the “85th percentile” critical point, risk was classified into three levels according to the intensity of the bullying (mild, moderate, and severe).

Some research studies do, however, show a gender difference, greater among boys than among girls during adolescence [55,56]. But other studies report contrary results, indicating that both girls and boys bully, and that the difference lies in the visibility of their behaviour—more direct in boys (physical and verbal aggression) and more relational in girls (isolation and rejection) [57].

Regarding the variance of the difference between public and private owned schools, the study by Garaigordobil et al. [58] shows there is an absence of differences between the two types of environment, and affirms that bullying occurs in all schools, regardless of their sociocultural and religious characteristics.

This study has shown that both the risk of being a victim of bullying and that of having emotional problems can be validly and reliably detected in a particularly sensitive age group (between 11 and 13 years old). The comparative risk tables allow a pupil to be placed at a certain level of risk in both aspects. Nonetheless, since the detection is carried out at a certain point in their schooling, it is necessary to be attentive to the possible changes that may occur in the pupils’ lives.

The four basic situations in which a pupil might find themself are described in the following simplified form:

*Situation 1:* No risk of bullying and no risk of emotional problems (−B/−E), the most frequent situation (73.2%). The pupils in this group show no risk of bullying or emotional problems.

*Situation 2:* At risk of bullying but without risk of emotional problems (+B/−E), with 11.1% of the pupils falling into this group. These pupils do not show emotional suffering, although it is necessary to provide them with personal strategies to deal with possible bullying.

*Situation 3:* No risk of bullying but with risk of emotional problems (−B/+E), with 9.4% of the pupils in this group. Since these pupils do not perceive themselves as being a victim of bullying, their emotional problems may not be related to this aspect but to others of a contextual and/or personal nature. In this case, it would be recommendable to deepen into knowledge of the underlying causes provoking their emotional situation so as to plan the necessary help.

*Situation 4:* At risk of bullying and at risk of emotional problems (+B/+E). This is the most serious case and the least frequent (6.3%). and it is necessary to implement the actions required to combat it. Prevention is no longer enough, and the subject clearly perceives themself to be a victim, which is aggravated by emotional problems which may or may not derive from the possible bullying. Given this situation, we recommend implementing the intervention protocol given the detected risk, with the prudence that should accompany these interventions. A first step is to open up an information file to gain depth into the pupil’s personal and social situation: who is (or are) the alleged aggressor(s), where it occurs and at what time, what type of aggression is suffered (verbal, physical, psychological, …), how the presumed victim feels, and what personal resources they have to deal with their situation.

## 5. Conclusions

This study has adapted and verified the reliability and validity of a joint questionnaire to detect the risk of bullying and emotional problems in pupils from 11 to 13 years old. It was found that the most frequent actions of bullying were those related to verbal aggression (insults, name-calling, and speaking ill of the victim), while the least frequent actions were serious aggressions (threatening with weapons, sexual harassment, and using threats to force the victim to do things), as is logical considering the age of the pupils. With respect to emotional problems, high scores in psychosomatic aspects (tiredness, sleeping difficulties, tingling sensations of “pins-and-needles”, …) and feelings of uselessness, uncertainty, and sadness were the main characteristics perceived.

In summary, we obtained a simple and practical instrument that can be recommended for use in schools and health services for children between 11 and 13 years old, and its use for older adolescent pupils is not ruled out. In both cases, it is useful to have this information in order to plan actions and implement programs to reduce the frequency of bullying and the suffering of the victims and their families.

One of the limitations of the study is the narrow age range of the sample. It would therefore be advisable to continue with the expansion of the samples, considering that it would be suitable for ages from 11 to 16, the end of primary education and all of lower (compulsory) secondary education. Including questions related to cyberbullying would also complete the study. We would recommend the use of computer resources to collect information through online questionnaires in schools. This would greatly facilitate data and table coding for later analysis.

Also, the fact of the data being self-reported requires the limitations of that technique to be taken into account: in particular, “false positives”, i.e., those who perceive themselves as victims without objectively being so, and “false negatives”, those pupils at risk of being victims but who do not perceive themselves as such. For this reason, the use of other complementary techniques such as teachers’ observation of their pupils’ behaviour, interviews, or other questionnaires addressed to observers might be capable of completing the detection.

## Figures and Tables

**Table 1:** Community Ratio Indices and KMO values.

	Bullying Victim		Emotional Problems	
	Subsample 1	Subsample 2	Subsample 1	Subsample 2
CRI	0.979		0.999	
KMO	0.877	0.858	0.924	0.923

**Table 2:** Main descriptive statistics of the bullying victim detection scale.

ITEMS	Mean	SD	MSA	Sk	Ku
They insult me	1.54	0.774	0.936	1.519	2.001
They call me names	1.53	0.822	0.928	1.652	2.111
They bad-mouth me	1.50	0.749	0.927	1.678	2.692
They hide things from me	1.34	0.644	0.862	2.170	4.862
They ignore me	1.28	0.561	0.782	2.304	6.119
They won’t let me participate	1.23	0.560	0.704	2.899	9.175
They steal things from me	1.16	0.444	0.899	3.298	12.518
They hit me	1.15	0.469	0.809	3.623	14.737
They threaten me (to scare me)	1.15	0.474	0.918	3.690	15.172
They break my things	1.11	0.379	0.889	4.219	21.895
They sexually harass me	1.03	0.209	0.944	7.717	62.854
They use threats to make me do things	1.03	0.236	0.918	8.600	86.759
They threaten me with weapons	1.02	0.191	0.883	9.462	103.780

**Table 3:** Main descriptive statistics of the emotional problems detection scale.

ITEMS	Mean	SD	MSA	Sk	Ku
I wake up in the morning tired	2.67	1.471	0.910	0.325	−1.263
I get very sleepy during the day	2.21	1.259	0.911	0.832	−0.291
I’m scared people will make fun of me	2.00	1.281	0.957	1.105	0.058
I get numbness in my arms, hands, etc.	1.83	1.031	0.897	1.140	0.692
I’m scared I won’t know how to do the homework well	1.83	1.116	0.958	1.302	0.901
I have a hard time going to sleep	1.79	1.140	0.953	1.441	1.197
I get pins-and-needles in parts of my body	1.68	1.030	0.946	1.594	1.927
I think I don’t do anything right	1.68	1.021	0.959	1.537	1.752
I feel sad	1.64	0.977	0.958	1.595	2.057
I want to be alone	1.61	1.009	0.975	1.727	2.384
My hands tremble	1.60	0.952	0.953	1.638	2.152
I think there’s nothing I’m of use for	1.55	1.013	0.932	1.941	3.054
I think I’m worthless	1.44	0.921	0.934	2.280	4.688
I’m no good for anything	1.43	0.916	0.932	2.299	4.468
I feel very lonely	1.39	0.884	0.955	2.515	5.998
I feel empty	1.34	0.827	0.940	2.712	7.109

**Table 4:** Structure matrix of the bullying detection scale.

ITEMS	Factor 1	Factor 2	Factor 3
They steal things from me	0.841		
They use threats to make me do things	0.760		
They break my things	0.719		
They hide things from me	0.693		
They threaten me with weapons		0.997	
They sexually harass me		0.562	
They insult me			0.852
They ignore me			0.791
They bad-mouth me			0.780
They threaten me (so as to scare me)			0.778
They hit me			0.762
They call me names			0.746
They won’t let me participate			0.702

**Table 5:** Structure matrix of the emotional problems detection scale.

ITEMS	Factor 1	Factor 2
I wake up in the morning tired	0.663	
My hands tremble	0.632	
I have a hard time going to sleep	0.613	
I get pins-and-needles in parts of my body	0.595	
My arms, hands, etc. go numb	0.582	
I get very sleepy during the day	0.582	
I think I’m worthless		0.910
I’m good for nothing		0.859
I think there’s nothing I’m of use for		0.845
I feel very lonely		0.825
I feel empty		0.818
I think I don’t do anything right		0.810
I feel sad		0.784
I’m scared people will make fun of me		0.695
I want to be alone		0.665
I’m scared I won’t know how to do the homework well		0.651

**Table 6:** Comparison of means according to gender.

	Bullying Victim		Emotional Problems	
	Man (*n* = 549)	Woman (*n* = 528)	Man (*n* = 549)	Woman (*n* = 528)
Arithmetic mean	16.41	15.72	26.88	28.53
SD	4.545	3.450	10.259	10.534
M-W U-test *z* value	−1.499 (*p =* 0.134)		−3.162 (*p =* 0.002)	
Effect size (Cohen’s *d*)	0.17		0.16	
Statistical power (1-*β*)	1.00		0.72	

**Table 7:** Comparison of means according to education level.

	Bullying Victim		Emotional Problems	
	6th Primary (*n* = 467)	1st ESO (*n* = 610)	6th Primary (*n* = 467)	1st ESO (*n* = 610)
Arithmetic mean	16.31	15.89	27.76	27.63
SD	4.179	3.957	10.450	10.410
M-W U-test *z* value	−1.747 (*p =* 0.081)		−0.289 (*p =* 0.773)	
Effect size (Cohen’s *d*)	0.10		0.01	
Statistical power (1-*β*)	0.49		1.00	

**Table 8:** Comparison of means according to the ownership of the school.

	Bullying Victim		Emotional Problems	
	Public (*n* = 899)	Private (*n* = 178)	Public (*n* = 899)	Private (*n* = 178)
Arithmetic mean	16.07	16.10	27.73	27.50
SD	4.079	3.966	10.520	9.941
M-W U-test *z* value	−0.163 (*p =* 0.871)		−0.017 (*p =* 0.986)	
Effect size (Cohen’s *d*)	0.01		0.02	
Statistical power (1-*β*)	0.05		0.06	

**Table 9:** Overall descriptive statistics for the bullying and emotional problems detection scales.

	Bullying Victim Detection Scale	Emotional Problems Detection Scale
Arithmetic mean	16.08	27.69
SD	4.058	10.422
Median	15.00	25.00
Standard error of the mean	0.124	0.318
Skewness	2.438	1.410
Kurtosis	8.227	2.299
K-S (normality) Centiles 25-50-75	0.224 (*p* < 0.001) 13-15-17	0.131 (*p* < 0.001) 20-25-33

**Table 10:** Risk levels from raw scores.

Risk Level	Direct Scores	
	Bullying Victim Detection Scale	Emotional Problems Detection Scale
Severe risk (≥95th centile)	24 and over	50 and over
Moderate risk (90–94th centile)	21–23	43–49
Mild risk (85–89th centile)	19–20	39–42
No risk (<85th centile)	18 and under	38 and under

**Table 11:** Risk of emotional problems and the mean scores on the bullying scale.

		EMOTIONAL P.	
BULLYING VICTIM	No. (%)	Mean	SD
Severe risk (95th centile and above)	60 (5.6)	38.17	13.519
Slight-moderate risk (85–94 centile)	128 (11.9)	34.16	12.155
No risk (<85th centile)	889 (82.5)	26.05	9.066
Total	1077 (100%)	27.69	10.422

**Table 12:** Risk of being a victim of bullying and the mean scores on the emotional problems scale.

		EMOTIONAL P.	
BULLYING VICTIM	No. (%)	Mean	SD
Severe risk (95th centile and above)	60 (5.6)	38.17	13.519
Slight-moderate risk (85–94 centile)	128 (11.9)	34.16	12.155
No risk (<85th centile)	889 (82.5)	26.05	9.066
Total	1077 (100%)	27.69	10.422

## Data Availability

Not applicable.
